# Can paternal leakage maintain sexually antagonistic polymorphism in the cytoplasm?

**DOI:** 10.1111/jeb.12582

**Published:** 2015-02-27

**Authors:** B Kuijper, N Lane, A Pomiankowski

**Affiliations:** * CoMPLEX, Centre for Mathematics and Physics in the Life Sciences and Experimental Biology, University College LondonLondon, UK; † Department of Genetics, Evolution and Environment, University College LondonLondon, UK

**Keywords:** chloroplast, heteroplasmy, intralocus sexual conflict, maternal inheritance, mitochondria, mtDNA, organelle, sexual dimorphism

## Abstract

A growing number of studies in multicellular organisms highlight low or moderate frequencies of paternal transmission of cytoplasmic organelles, including both mitochondria and chloroplasts. It is well established that strict maternal inheritance is selectively blind to cytoplasmic elements that are deleterious to males – ’mother's curse’. But it is not known how sensitive this conclusion is to slight levels of paternal cytoplasmic leakage. We assess the scope for polymorphism when individuals bear multiple cytoplasmic alleles in the presence of paternal leakage, bottlenecks and recurrent mutation. When fitness interactions among cytoplasmic elements within an individual are additive, we find that sexually antagonistic polymorphism is restricted to cases of strong selection on males. However, when fitness interactions among cytoplasmic elements are nonlinear, much more extensive polymorphism can be supported in the cytoplasm. In particular, mitochondrial mutants that have strong beneficial fitness effects in males and weak deleterious fitness effects in females when rare (i.e. ’reverse dominance’) are strongly favoured under paternal leakage. We discuss how such epistasis could arise through preferential segregation of mitochondria in sex-specific somatic tissues. Our analysis shows how paternal leakage can dampen the evolution of deleterious male effects associated with predominant maternal inheritance of cytoplasm, potentially explaining why ’mother's curse’ is less pervasive than predicted by earlier work.

## Introduction

Strict maternal inheritance of the cytoplasm is considered the norm in most higher eukaryotes. However, a growing number of studies in plants and animals report detectable levels of paternal transmission of cytoplasmic elements (e.g. Gyllensten *et al*., 1991; Schwartz & Vissing, 2002; Wolff *et al*., 2008; Nunes *et al*., 2013; Wolff *et al*., 2013; reviewed in Mogensen, 1996; McCauley, 2013). In some taxa, the rate reported is very low, for example 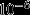 offspring in mice carry paternally inherited mitochondria (Gyllensten *et al*., 1991). But considerably higher levels of leakage occur in others: for example, 3–6% of seeds in *Silene vulgaris* bear paternal mtDNA (McCauley, 2013), and *Drosophila melanogaster* has recently been inferred to have levels of leakage of up to 6% (Nunes *et al*., 2013). Moreover, extreme examples of paternal mtDNA transmission in higher eukaryotes are found in sequoias (*Sequoia sempervirens*) and cucumber (*Cucumis sempervirens*), in which mitochondria are predominantly inherited through pollen, instead of ova (Neale *et al*., 1989; Havey, 1997; Crosby & Smith, 2012).

The findings above question whether conventional predictions about the evolutionary dynamics of cytoplasmic elements are robust to observed levels of paternal leakage (Wolff *et al*., 2013). One well-established idea is that cytoplasmic elements such as mitochondria, chloroplasts or endosymbionts invariably accumulate mutations that are detrimental to males (’mother's curse’, Frank & Hurst, 1996; Gemmell *et al*., 2004). This is because strict maternal inheritance masks any selection on male fitness effects, leading to the spread of sexually antagonistic alleles that are favoured in females but detrimental to males. Indeed, indirect evidence for mother's curse comes from a recent study by Innocenti *et al*. (2011), which shows that the introgression of a novel mitochondrial variant substantially affects male-specific gene expression, whereas female-specific gene expression remains unaffected. Moreover, a substantial number of mitochondrial mutations seem to exclusively affect male fitness, particularly in the context of male fertility (e.g. Nakada *et al*., 2006; Wai *et al*., 2010; Innocenti *et al*., 2011). One prominent example is Leber's disease, in which a mitochondrial mutation is responsible for loss of vision, which predominantly affects young males (Wallace *et al*., 1988). On the other hand, recent evaluations of mitochondrial disease in humans show that the majority of mtDNA mutations lack sex-specific effects (e.g. Park & Larsson, 2011; Wallace, 2013), begging the question whether other mechanisms exist that mitigate the extent of mother's curse (e.g. Wade & Brandvain, 2009).

Here, we ask whether small amounts of leakage affect cytoplasmically linked detrimental male fitness effects. To assess sexually antagonistic variation in the cytoplasm, we use a two-fold approach. First, we incorporate leakage in the population genetics model of Frank & Hurst (1996). This model implicitly assumes a rather simplistic form of mitochondrial inheritance, where all cytoplasmically linked alleles within an individual are identical. By contrast, studies of the dynamics of mitochondrial transmission (Birky, 1995, 2001; Stewart *et al*., 2008a) emphasize that bottlenecks, sampling effects and vegetative segregation are important, which potentially allow for heteroplasmy, in which individuals carry more than one cytoplasmically linked allele (Hadjivasiliou *et al*., 2012, 2013). At present, no predictions exist about how the interactions between multiple cytoplasmic alleles affect fitness and sexually antagonistic polymorphism. These interactions are likely to be important as epistatic and dominance interactions clearly affect the scope for sexually antagonistic variation on autosomes (Kidwell *et al*., 1977; Rice, 1984; Fry, 2010; Connallon & Clark, 2011; Mullon *et al*., 2012). We therefore assess the consequences of different types of epistatic interactions within the cytoplasm for sexually antagonistic variation in the presence of leakage.

The main goal of the current model was to investigate whether slight levels of leakage could provide an additional explanation for the limited prevalence of mother's curse in nature. In this context, a previous model on mitochondrial effects on male fertility (Wade & Brandvain, 2009) verbally alludes to the potential of leakage to reduce mother's curse. A systematic analysis of the effects of leakage on mother's curse is lacking for the broad range of leakage values that have been found. Another goal was to quantify how leakage affects heteroplasmy (McCauley, 2013), particularly when the fitness consequences of cytoplasmic genes diverge between the sexes. Although classical studies on the maintenance of genetic variation in the cytoplasm have considered leakage (e.g. Ohta, 1980; Takahata & Maruyama, 1981; Takahata & Slatkin, 1983), the role of sex-specific fitness effects and interactions between multiple cytoplasmic alleles within an individual have not been considered previously.

## Model 1: haploid population genetics

In the remainder of this article, we refer to mitochondria as the cytoplasmic element in question, as they are the most common and ancient cytoplasmic element in nature (Lane & Martin, 2010; Martin, 2011). However, we emphasize that similar principles hold for other cytoplasmic elements (e.g. chloroplasts and endosymbionts such as *Wolbachia*). We first focus on paternal leakage in the population genetics model studied by Frank & Hurst (1996), in which all mitochondrial alleles are identical within an individual. We consider the fate of two mitochondrial alleles, 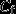 being a female-benefit, male-detriment allele, whereas 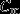 is a male-benefit, female-detriment allele. We track the frequency of the 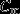 allele in females (frequency 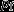) and males (frequency 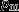). Similar to Frank & Hurst (1996), females bearing a 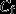 or a 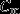 allele have survival probabilities of 1 and 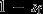, respectively, whereas males have corresponding survival probabilities 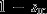 (allele 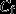) and 1 (allele 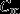) (see Table 1). Frequencies of the 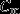 allele in females and males in the next generation are then given by


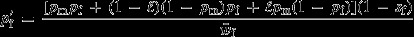
(1a)


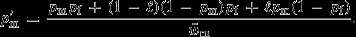
(1b)

**Table 1:** Sex-specific fitness functions used in the haploid model and the model involving multiple mitochondria. 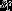 and 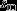 reflect the baseline mortality cost of bearing 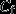 and 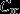 alleles. *M* reflects the total number of mitochondria present in an individual, *m* of which are of type 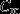 and *M* − *m* are of type 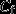. *k* reflects how rapidly fitness increases (or decreases) with an increasing number of 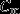 mitochondria

Model	Context	Female fitness	Male fitness
Haploid model	Female-benefit cytoplasm 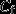	1	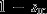
	Male-benefit cytoplasm 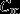	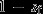	1
Multiple mitochondria	Constant dominance	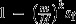	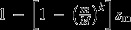
	Reverse dominance	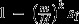	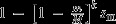
	Sigmoidal fitness	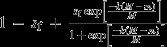	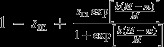

For 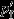, the first term within the straight brackets reflects the probability that daughters inherit a 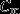 mitotype from a mating between a mother bearing the 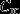 mitotype (with frequency 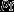) and a 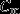 father (frequency 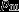). In this case, leakage does not affect transmission, as both parents carry the 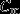 mitotype. Daughters also inherit the 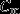 allele when a 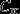 mother mates with a 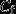 father and leakage does not occur with probability 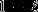 (second term), or when a 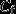 mother mates with a 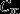 father and leakage occurs with probability 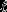 (third term). In all cases, daughters that inherit the 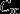 allele have a fitness of 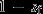. Similar considerations govern the inheritance of 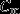 by sons, except that sons who inherit the 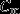 allele have a fitness of unity. Expressions for female and male mean fitness are given by


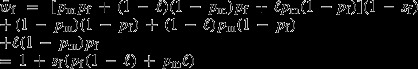
(2a)


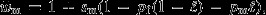
(2b)

yielding the corresponding difference equations 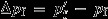 and 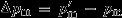. Solving for 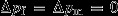 yields three equilibria: one in which 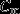 is extinct (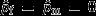), one where 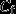 is extinct (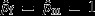) and an equilibrium where 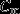 and 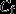 coexist:


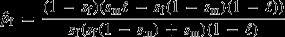
(3a)


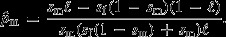
(3b)

By evaluating the eigenvalues of the Jacobian matrix of the system in eqns (1a and 1b), we then assess how paternal leakage affects the stability of these equilibria (see below).

### Result 1: mitochondrial polymorphism is only maintained when selection on males is strong

**Figure 1:**
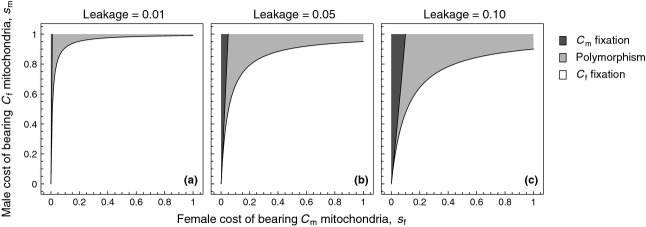
Regions depicting the effect of leakage on the maintenance of 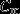 mitochondria when individuals contain a single, haploid mitochondrion. Regions depict fixation of female-benefit 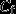 mitochondria (white), polymorphism of 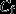 and male-benefit 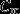 mitochondria (light grey), and fixation of 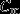 mitochondria (dark grey).

Consider a population that is fixed for 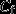, the female-benefit, male-detriment mitochondrial allele (i.e. 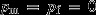). A rare mutant mitochondrion 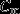 that benefits males but is detrimental to females invades when


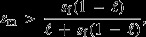
(7)

which reduces to the classical condition 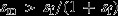 (Kidwell *et al*., 1977) for autosomal alleles (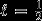). Unsurprisingly, when leakage is absent 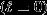, we find that 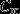 is unable to invade unless 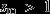, in which case no male survives and the population goes extinct. However, 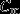 is able to invade for modest levels of leakage 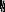 when the fitness effect of 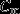 in females is modest relative to the deleterious fitness effects of the ’resident’ mitochondrion 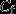 on males. Upon invasion, 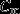 and 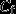 are both stably maintained in an equilibrium given by eqns (3a and 3b) when


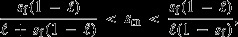
(8)

which is identical to the conditions for polymorphism identified by Kidwell *et al*. (1977) for autosomal loci 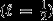 in the absence of dominance. Both conditions (7) and (8) are graphically summarized in Fig. 1 for realistic levels of leakage observed in natural populations. We conclude that, for modest levels of leakage 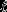, mitochondrially linked male-benefit mutations will be unable to invade for a large region of the parameter space. This formalizes a previous verbal prediction by Wade & Brandvain (2009) (pp. 1087–1088) that low levels of leakage are unlikely to affect mitochondrial sexually antagonistic variation when selection is relatively weak. However, when selection on males is strong, we find that invasion of 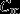 typically results in the stable maintenance of both 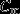 and 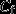 mitotypes (i.e. polymorphism). Lastly, for a much narrower range of the parameter space where leakage is considerable 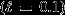 and selection on females is very weak relative to selection on males 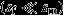, we find that cytotypes that are deleterious for females are able to fix in the population.

## Model 2: multiple mitochondrial alleles per individual

Until now, we assumed that each individual contains only one mitochondrial allele. However, cells usually contain multiple mitochondria and multiple mtDNA molecules within each mitochondrion (Birky, 2001). Taking account of multiple alleles is essential given that paternal leakage is likely to result in heteroplasmy, in which different mitochondrial alleles coexist in a single individual (McCauley, 2013). We therefore investigate a model that allows for the presence of a finite number of *M* mitochondrial alleles within an individual (see also Hadjivasiliou *et al*., 2012). The dynamics of the number of cytoplasmic alleles per individual is then subject to a range of additional forces, including sampling due to drift (Ohta, 1980; Chapman *et al*., 1982), bottlenecks (Bergstrom & Pritchard, 1998), vegetative segregation (Birky, 2001), as well as selection arising from the epistatic interactions between different alleles within an individual.

**Figure 2:**
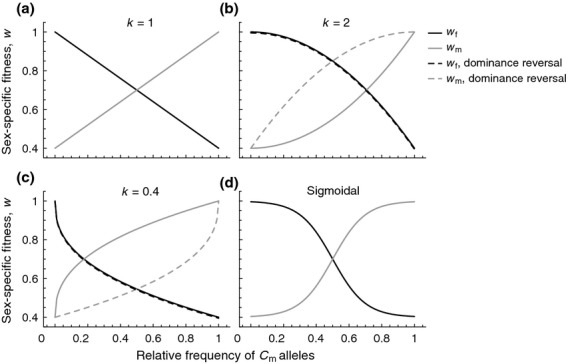
Male and female fitnesses when individuals contain more than a single cytoplasmic element. For all panels, solid lines indicate a scenario of ’constant dominance’ where the fitness gain in one sex is exactly mirrored by the fitness loss in the other sex. Dashed lines indicate a scenario of ’reverse dominance’, where the fitness gain in one sex is different from the fitness loss in the other sex. (a) *k* = 1, sex-specific fitness changes linearly with the proportion of 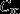 mitochondria in both males and females. (b, c) sex-specific fitness changes either in an accelerating or decelerating fashion with an increasing proportion of 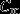 mitochondria. See Table 1 for formal descriptions of the different fitness functions. (d) sex-specific fitness changes in a sigmoidal fashion. Parameters for all panels: 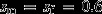. Panel (d) *k* = 0.1.

*Fitness*: To model the effect of epistatic interactions between 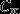 and 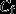 mitochondria on sexually antagonistic variation, let *m* be the number of 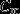 mitochondria present in an individual (and *M*−*m* the number of 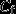 mitochondria). Following previous multilocus models that deal with sexual antagonism (e.g. Connallon & Clark, 2011; Hadjivasiliou *et al*., 2012), we assume that female and male fitnesses are then dependent on the proportion of male-benefit versus female-benefit alleles (see Table 1):


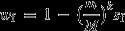
(6a)


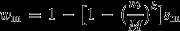
(6b)

where *k* reflects the type of epistatic relationship between multiple mitochondrial alleles present in a single individual, in a similar fashion as dominance interactions have been modelled between alleles at a single locus (see also Fry, 2010; Connallon & Clark, 2011). When *k* = 1, mitochondria interact additively (see Fig. 2a). By contrast, when *k* > 1, 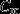 mitochondria are recessive to 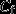 (solid lines in Fig. 2b) and when *k* < 1, 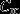 mitochondria are dominant relative to 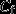 (solid lines in Fig. 2c). Importantly, the dominance of 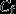 relative to 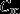 is the same across males and females (i.e. a ’constant dominance’ scenario; Connallon & Clark, 2011). Another way of thinking about these assumptions is that when 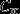 is dominant, there is strong fitness loss in females and strong fitness gain in males when 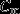 is at low frequency within an individual (solid lines in Fig. 2c). When 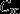 is recessive, the fitness loss in females and fitness gain in males is weak when 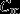 mitochondria are at low frequency (solid lines in Fig. 2). The fitness gain in one sex is exactly mirrored by the fitness loss in the other sex.

Alternatively we consider a fitness function for ’reversed dominance’ (e.g. Curtsinger *et al*., 1994; Connallon & Clark, 2011), where the dominance of 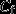 relative to 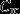 varies according to the sex of the carrier. Male fitness is now given by: 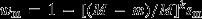 instead of eqn (6b), whereas female fitness is the same as in eqn (6a) (see Table 1). For example, when *k* > 1, a modest fitness loss in females could be mirrored by a strong fitness gain in males when 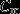 mitochondria are at low frequencies (dashed lines in Fig. 2b). Similarly, a strong fitness loss in females could be mirrored by weak fitness gain in males when the 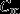 allele is at low frequency when *k* < 1 (dashed lines in Fig. 2c).

Lastly, we also assess a more complex fitness function, where 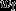 and 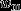 are given by a sigmoidal function (see Fig. 2d). Such a sigmoidal function reflects not only sexual antagonism, but also scenarios where the presence of polymorphism in 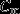 and 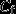 itself substantially reduces viability, conforming to recent findings that heteroplasmy (which reflects within-individual polymorphism) negatively affects fitness in certain cases (Lane, 2012; Sharpley *et al*., 2012; Wallace & Chalkia, 2013).

**Figure 3:**
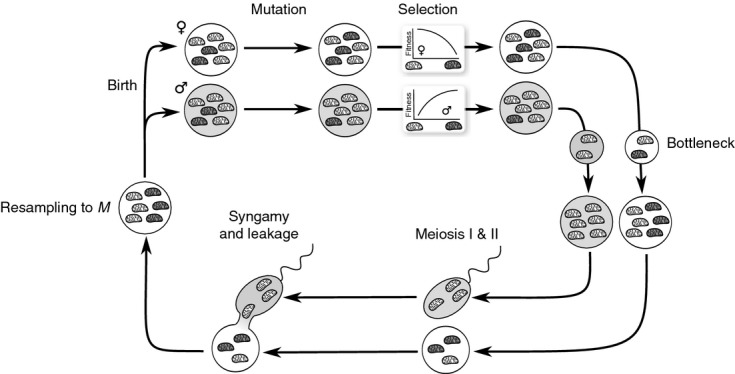
Schematic depiction of the life cycle when individuals contain multiple mitochondria.

*Life cycle*: To model change in allele frequencies in the presence of multiple cytoplasmic elements, we assume a life cycle similar to that used by Hadjivasiliou *et al*. (2012) to model the evolution of uniparental inheritance of mitochondria (which we refer to for an in-depth discussion). The life cycle consists of five phases (see SI and Fig. 3): (i) at birth, an individual contains *M* cytoplasmic elements, (ii) individual mitochondria mutate independently of each other with probability *μ*, (iii) individuals then undergo sex-specific selection according to the equations in Table 1, (iv) surviving individuals enter a sexual phase in which they undergo meiosis and (v) syngamy to produce the next generation. Before meiosis, individuals undergo a bottleneck in which *B* mitochondria are sampled from the *M* original mitochondria (Birky, 1995, 2001), after which the population size of mitochondria is brought back up to *M* by randomly sampling with replacement from the *B* mitochondria. Although it is currently debated whether within-host purifying selection or bottlenecks are the underlying cause of rapid mtDNA segregation in animals (Stewart *et al*., 2008b; Wai *et al*., 2008; Samuels *et al*., 2010; Carling *et al*., 2011), the goal of the bottleneck stage is to assess the effect of within-host homogenization of cytoplasmic elements. After the bottleneck stage, each cell that undergoes meiosis produces 2*M* mitochondria, which are then reduced through two cell divisions to *M*/2 mitochondria present in each of the four gametes. Gametes then randomly fuse with each other, where the female gamete contributes 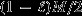 mitochondria (where 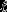 is the proportion of leakage), and the male gamete contributes 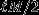 mitochondria. The resulting *M*/2 mitochondria are then doubled through sampling with replacement to arrive back at *M* mitochondria. The life cycle can be mathematically described in an exact manner, but the complexity of the model prohibits an analytical solution. We therefore use numerical simulations to find equilibrium frequencies of cytotypes 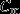 and 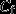. For the sake of computational speed, we consider a conservative value of *M* = 200 copies of cytoplasmic genomes per cell, which is typically the lower bound of the number of mitochondrial copies in animals (e.g. Solignac *et al*., 1984; Cao *et al*., 2007). Moreover, a small value of *M* in combination with the aforementioned bottleneck is indicative of the fact that mitochondria typically have small effective population sizes (Ballard & Whitlock, 2004; Neiman & Taylor, 2009).

### Result 2: cytoplasmic epistasis favours polymorphism and heteroplasmy

**Figure 4:**
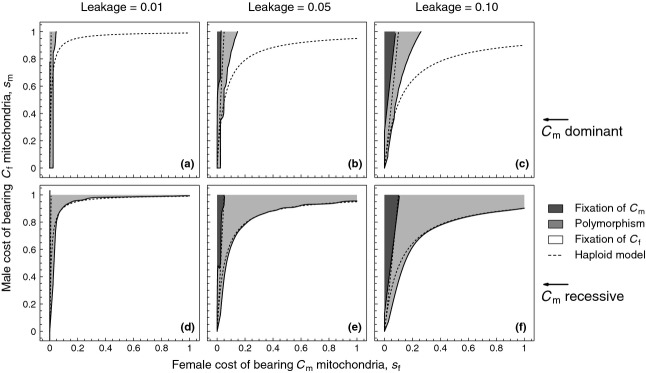
Cytoplasmic polymorphism for different levels of leakage 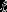. Fitness effects are assumed to be nonlinear and felt similarly by males and females (see solid lines in Fig. 2a,b). Panels (a–c) fitness effects of a rare 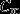 allele are dominant in both sexes (*k* = 0.25). Panels (d–f) fitness effects of a rare 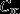 allele are recessive in both sexes (*K*=2). Parameters: *M* = 200, 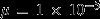. Polymorphism is defined as 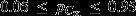. 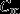 is considered fixed when 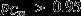.

For individuals with multiple cytoplasmic elements (*M* = 200), polymorphism does not differ from the haploid model (where *M* = 1, see Fig. 1) when fitness effects are additive (see Fig. S1). Similar to the haploid model, higher levels of paternal leakage (large 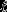) together with strong selection on males (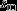 large) facilitate the presence of cytoplasmic polymorphism. By contrast, when fitness effects are nonlinear and felt similarly by males and females (solid lines in Fig. 2b and c), the degree of polymorphism differs. When 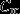 mitochondria are dominant in both sexes, they are much less likely to be maintained (Fig. 4a–c). However, when 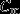 mitochondria are recessive in both sexes, differences with the haploid model are small (Fig. 4d–f). Likewise for sigmoidal nonadditive interactions (Fig. 2d), we find that the parameter space conducive to polymorphism is only slightly enhanced relative to the haploid model (see Fig. S2).

**Figure 5:**
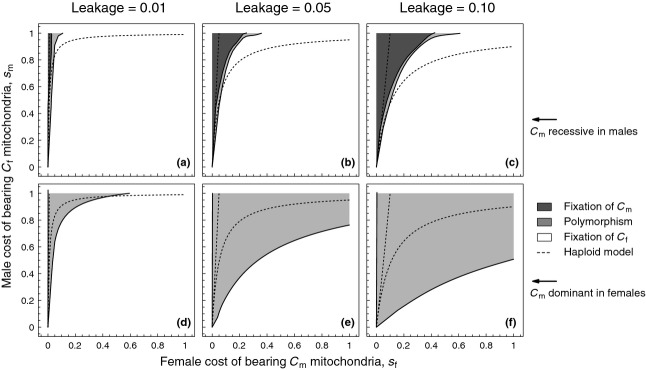
Cytoplasmic polymorphism for different levels of leakage 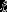. Epistatic interactions among cytoplasmic elements reflect a scenario of ’reverse dominance’ (dashed lines in Fig. 2b, c). Panels (a–c) rare 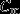 mitochondria are recessive in males and dominant in females (*k* = 0.25). Panels (d–f) rare 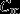 mitochondria are dominant in males and recessive in females (*k* = 2). Parameters: *M* = 200, 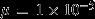. Polymorphism is defined as 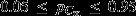. 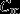 is considered fixed when 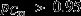.

The conditions leading to sexually antagonistic polymorphism are strikingly different from the haploid model in the case of reverse dominance (Fig. 5). When the male-beneficial allele 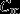 is recessive in males and hence dominant in females, we find that the region of polymorphism is markedly smaller relative to the haploid model (Fig. 5a–c). When 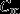 successfully invades, it nearly always achieves fixation, as the fitness benefit to males increases more than linearly the more common it gets (dashed line for 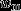 and solid line for 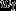 in Fig. 2c). By contrast, when the male-beneficial allele 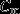 is dominant in males and recessive in females, we find that 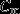 can invade for considerably lower values of 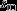 relative to the haploid case ( Fig. 5d–f). The 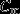 allele invades as its fitness when rare is largely masked in females, whereas it provides a large fitness benefit in males. However, it will typically not achieve fixation, as the fitness cost for females (which transmit the majority of mitochondria) rises markedly with increasing frequency of 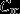 (dashed line for 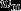 and solid line for 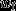 in Fig. 2b). Consequently, polymorphism is expected to occur in the presence of leakage for male-benefit alleles that are dominant in males, but recessive in females.

**Figure 6:**
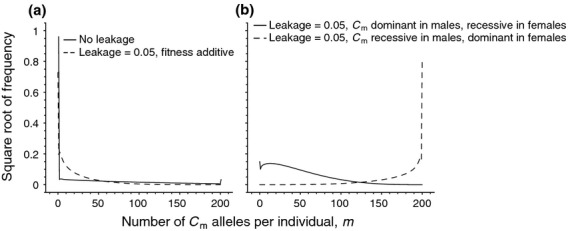
Example distributions of the frequency (square-root transformed) of individuals bearing *m*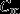-alleles. Panel (a) when leakage is absent or fitness effects are additive, heteroplasmy is nearly negligible and most individuals contain only 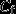 mitochondria. Panel (b) when leakage is present and fitness effects reflect a scenario of ’reverse dominance’ (dashed lines in Fig. 2b, c), levels of heteroplasmy can be substantial, particularly when 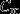 is dominant in males, but recessive in females (solid line). By contrast, when 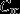 is recessive in males, yet dominant in females, levels of polymorphism are substantially lower. For the restrictive range of parameters for which 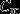 invades (when selection on males is very strong), it will achieve fixation (dashed line). Parameters throughout: 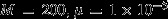; panel (a) 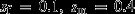; dashed line: 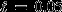. Panel (b) 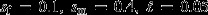 (solid line). 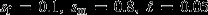 (dashed line).

The degree of polymorphism is also reflected by heteroplasmy, the variation of mitochondria within an individual. For a null scenario without any leakage and additive fitness effects (solid line in Fig. 6a), heteroplasmy is only caused by recurrent mutation and increases in the frequency of 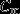 alleles are due to drift. Consequently, the majority of individuals only bear 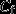 mitochondria, whereas heteroplasmic individuals are very rare and a small fraction of individuals are fixed for 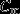. When leakage is nonzero and fitness is additive, we find a slight increase in heteroplasmy (dashed line in Fig. 6a). Most individuals are still homogeneous for 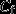 mitochondria, however, as selection on males is not strong enough to favour much polymorphism when fitness is additive. As a result, the overall frequency of 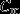 mitochondria does not exceed our threshold of *P* = 0.05 that demarcates the zone of polymorphism (Fig. S2).

The scope for higher frequencies of 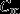 alleles is enhanced with reverse dominance ( Fig. 6b). The selection pressure when the 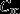 allele is dominant in males and hence recessive in females is now strong enough to lead to high levels of heteroplasmy (solid line in Fig. 6b). This is not true in the opposite case when the 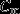 allele is recessive in males and dominant in females, as selection in favour of 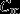 tends to lead to a situation where most individuals are fixed for 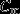 mitochondria (dashed line in Fig. 6b).

**Figure 7:**
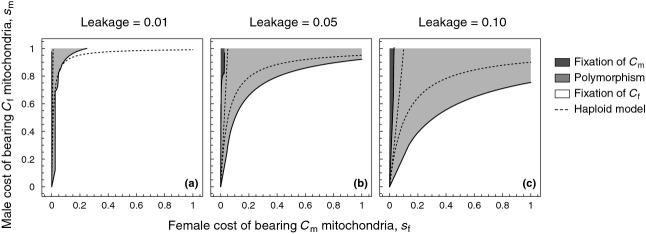
The occurrence of cytoplasmic polymorphism for different levels of leakage 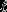, when multiple cytoplasmic elements are present per individual and a bottleneck *B* = 10. Epistatic interactions among cytoplasmic elements reflect a scenario of ’reverse dominance’ in which 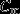 mitochondria in males are dominant over 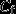 mitochondria (*k* = 2), similar to Fig. 5d–f. Parameters: *M* = 200, 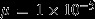.

Finally, we consider how mechanisms that increase within-individual homogeneity of mitochondria (e.g. bottlenecks) impact on the parameter space in which polymorphism is found under paternal leakage. Considering an illustrative bottleneck size of *B* = 10, we find that the domain of polymorphism is hardly affected when fitness effects are additive (Fig. S3) or 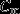 is dominant (Fig. S4). However, under reverse dominance in which the area favouring polymorphism is greater (Fig. 5d–f), we find that results are sensitive to within-individual homogenization of mitochondria due to bottlenecks (Fig. 7), reducing the area favouring polymorphism.

## Discussion

The matrilineal transmission of cytoplasmic elements – such as mitochondria or endosymbiotic bacteria like *Wolbachia* – is widely understood to lead to mother's curse (Frank & Hurst, 1996; Gemmell *et al*., 2004), where selection cannot stop the build-up of cytoplasmic mutations that are detrimental to males. Given the importance of mitochondria for male metabolism, we would expect mother's curse to be severe. Indeed, a number of examples exist that are in line with mother's curse (Camus *et al*., 2012; Yee *et al*., 2013; Beekman *et al*., 2014), but other studies have failed to find sex-specific fitness asymmetries of cytoplasmic elements (Friberg & Dowling, 2008; Schaefer *et al*., 2008; Mossman *et al*., 2010, 2012; Schon *et al*., 2012). A number of resolutions have been suggested to explain the limited occurrence of mother's curse, such as the presence of inbreeding and sperm limitation (Wade & Brandvain, 2009; Zhang *et al*., 2012) or recruitment of compensatory autosomal mutations to counter detrimental male fitness effects (Beekman *et al*., 2014). Here, we assess whether low levels of paternal mtDNA leakage – which have been recorded in an increasing number of species (McCauley, 2013) – could potentially provide another explanation for the limited generality of mother's curse.

The current analysis shows that for some of the fitness functions considered, haploid models of cytoplasmic inheritance (Frank & Hurst, 1996; Unckless & Herren, 2009; Wade & Brandvain, 2009) provide a robust description of cytoplasmic variation. This is particularly the case when fitness is linear or symmetric across the sexes (e.g. solid lines in Fig. 2a–d). In these cases, leakage only favours cytoplasmic mutations that benefit males when fitness effects are strong relative to fitness effects on females. This suggests that there is limited scope for paternal leakage as a mechanism to ameliorate mother's curse, given that selection coefficients measured in nature are generally weak (Kingsolver *et al*., 2001). However, when the shape of the fitness function differs between the sexes (dashed lines in Fig. 2b–d), we find that the haploid model fails. It is unable to capture the fine-grained costs and benefits of carrying a small number of 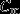 mitochondria (favoured in males but deleterious in females), or to predict the resulting levels of heteroplasmy, that in principle should be easily measured. This echoes previous work on the evolution of the sexes (Hadjivasiliou *et al*., 2012, 2013), which showed that taking account of multiple mitochondria provides a more subtle picture of the evolution of mitochondrial uniparental inheritance in comparison with previous studies that ignored the evolution of within-individual frequency of mutant mitochondria (Hurst & Hamilton, 1992; Randerson & Hurst, 1999).

Using this more sophisticated model to track multiple mitochondria per individual, leakage coupled to nonadditive fitness interactions leads to some plausible conditions that favour the spread of male-benefit mutations. In particular, when a rare mutant is dominant in males and recessive in females, a broader range of selective conditions lead to polymorphism (see dashed line in Fig. 2b). This effect is most prominent when the levels of leakage are relatively large (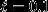), but polymorphism can still occur at lower levels of leakage (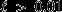). Current measured values of mtDNA leakage in animal and plant species are typically small, in the range between 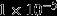 to 0.06 (Wagner *et al*., 1991; Svab & Maliga, 2007; Wolff *et al*., 2008; McCauley, 2013; Nunes *et al*., 2013). However, we are still far from a conclusive picture about the prevalence of leakage, as paternal inheritance of mtDNA has only been assessed for a limited number of species under a limited number of environmental conditions (reviewed in McCauley, 2013; Greiner *et al*., 2015). Chloroplast inheritance patterns appear to be more variable, with biparental inheritance of cpDNA found in 3 of 10 species measured so far (see Table 1 in Greiner *et al*., 2015). Consequently, more studies of leakage patterns in both mitochondria and chloroplasts in natural populations are likely to shed more light on the possibility of leakage leading to polymorphism.

The finding that polymorphism is particularly likely when male-benefit alleles impose a small fitness detriment on females, yet lead to large fitness benefits in males, begs the question whether this is likely in nature. One mechanism that could lead to such an asymmetric fitness landscape is the differential segregation of male- and female-benefit mitochondria to sex-specific tissues. For example, male-benefit mitochondria could segregate preferentially to male-specific reproductive tissues (e.g. sexual ornaments, parts of male genitalia not involved in gamete production, male muscular tissue, male parts of plants). Consequently, the majority of detrimental fitness effects of these male-benefit mitochondria would be masked in females (as they lack these tissues), while positively contributing to fitness in males, for example by allowing for a rate of mitochondrial biogenesis and energy metabolism closer to the male optimum for such tissues.

A mechanism based on preferential segregation of different mitochondrial types to different somatic tissues is supported by a number of recent studies using highly sensitive genetic techniques. These have shown that mitochondria in heteroplasmic individuals do indeed segregate in a tissue-specific fashion (Battersby *et al*., 2003; Magnacca & Brown, 2010; Burgstaller *et al*., 2014) and the frequency of mitochondrial mutants depends on tissue type (Dunbar *et al*., 1995; Chinnery *et al*., 1999; Samuels *et al*., 2013). For example, in heteroplasmic individuals from four species of Hawaiian bees, mitochondrial haplotypes present in the abdomen differ from those haplotypes in legs and thoracic flight muscle (Magnacca & Brown, 2010). Similar findings have been reported in bats (Brunet & Rossinni, 2004). Consequently, differential tissue segregation may be a plausible means through which the aforementioned fitness difference between the sexes can be achieved. Doubly uniparental inheritance in bivalve molluscs such as *Mytilus*, in which male-benefit mitochondria are specifically targeted to male gonads and female-benefit mitochondria to male somatic tissues, demonstrates that the segregation of mitochondria to male-specific tissues can and does occur (Breton *et al*., 2007; Zouros, 2013).

Differential tissue segregation need not be specific for male tissues if the fitness effects of male-benefit mitochondria are recessive in female tissues but dominant in male tissues with high energetic demand, including reproductive tissues. Given that males typically have higher metabolic demands (Mittwoch, 2004), one would predict that male-benefit mitochondria ought to be capable of sustaining a higher maximal metabolic rate; but that should not be especially detrimental to females. One possible female detriment is a greater leak of reactive oxygen species (ROS) from male-benefit mitochondria in female tissues (e.g. Ballard *et al*., 2007). If so, higher ROS leak could be diminished by either mild respiratory uncoupling (i.e. loss of coupling between respiration and ATP production; Lane, 2011b) or the production of relatively few male-benefit mitochondria, making them recessive in females. We would therefore expect to find higher rates of mitochondrial polymorphism or even fixation of male-benefit mitochondria in species with strong selection for male stamina.

We also showed that the severe homogenizing effect of bottlenecks in mitochondrial number could be important in those cases that favoured polymorphism. Bottlenecks are considered to be widespread in animals (Bergstrom & Pritchard, 1998; Stewart *et al*., 2008b; Lee *et al*., 2012), although the actual mechanism (i.e. the size of the bottleneck) that underlies this homogenization is still poorly understood (Carling *et al*., 2011). In plants, such homogenizing effects appear to be much rarer (Kmiec *et al*., 2006; Galtier, 2011, but see Morgan & Maliga, 1987). Consequently, the scope for cytoplasmically linked sexually antagonistic variation in the presence of leakage is likely to be substantially enhanced in plants relative to animals. However, as the literature on the size and cause of mitochondrial homogenizing effects in animals is scattered, mired in debate and focuses on a few species only (Carling *et al*., 2011; Wallace, 2013), it remains to be seen whether bottlenecks are the norm in animals.

Although considerable attention has been devoted to the study of interactions between cytoplasm and nucleus (e.g. Rand *et al*., 2004; Dowling *et al*., 2007a, 2008), little is known about epistatic interactions among the cytoplasmic elements themselves. In animals, studies of heteroplasmy have suggested that the mere presence of different types of mitochondria in a single individual can have negative fitness consequences (Wallace, 1994; Zeviani & Antozzi, 1997; Lane, 2011a; Lane, 2012; Sharpley *et al*., 2012). Again, plants appear to be the most amenable model organisms to test the fitness consequences of heteroplasmy, as substantial levels of heteroplasmy in both mtDNA (e.g. Städler & Delph, 2002) and chloroplast DNA (Frey *et al*., 2005) are found across a range of study systems (reviewed in McCauley, 2013). From our point of view, it would be particularly interesting to relate pollen and ovule production in plants to varying levels of heteroplasmy in cytoplasmic elements. Such experiments not only would allow one to assess whether different relative compositions of cytoplasmic elements have sex-specific effects, but also would provide measures of the epistatic fitness function (e.g. see Fig. 2). More generally, experiments like these are needed to assess whether selection on mitochondria is a general force rather than an exception (Ballard & Melvin, 2010).

Our study opens up several avenues for future work. Foremost, the current study considers leakage to be a fixed parameter, whereas it is likely to be a heritable character amenable to selection and evolutionary change. Modelling the evolution of leakage may shed light on the question of whether leakage is a sporadic aberration that mainly occurs in outbred or hybrid crosses (e.g. Fontaine *et al*., 2007; Hoarau *et al*., 2009; Hoolahan *et al*., 2011), or whether it is adaptive in its own right. Burt & Trivers (2006) suggested that paternal leakage is selectively favoured by the nucleus in the presence of cytoplasmic male sterility (CMS), as this favours symbionts that do not cause CMS (see also Wade & McCauley, 2005; McCauley, 2013). Even for male-detriment alleles with a less deleterious phenotype than CMS (e.g. Dowling *et al*., 2007b; Innocenti *et al*., 2011), we predict that the nucleus may favour a certain level of leakage, as accepting cytoplasmic elements from successful males ensures a lower degree of male-detriment effects in the cytoplasm. Nonetheless, leakage may also reintroduce costs that are associated with competition for replication between different cytoplasmic elements, for which sake uniparental inheritance may have evolved in the first place (Hurst & Hamilton, 1992). Consequently, whether leakage will indeed evolve needs to be addressed by formal studies. Models like these may also shed light on the striking variation in cytoplasmic inheritance patterns found in plants (Mogensen, 1996; McCauley, 2013), where maternal inheritance predominates, but multiple cases of paternal cytoplasmic inheritance (Neale *et al*., 1989; Havey, 1997) or biparental inheritance are known (Mogensen, 1996; Havey, 1997). All in all, the evolution of cytoplasmic inheritance and its consequences are still far from understood.

## Supporting information

**Figure S1** Cytoplasmic polymorphism with multiple mitochondria per individual and the shape of male and female fitness functions is given by a linear function (additive). **Figure S2** Cytoplasmic polymorphism when multiple mitochondria per individual are present and the shape of male and female fitness functions is given by a sigmoidal function (see Fig. 2d). **Figure S3** Cytoplasmic polymorphism when the shape of male and female fitness functions is additive and the size of the bottleneck *B* = 10. **Figure S4** Cytoplasmic polymorphism when the shape of male and female fitness functions is given by a scenario of constant dominance (solid lines in Fig. 2b and c) and the size of the bottleneck *B* = 10.

**Data S1** Model with multiple mitochondria.
