# Supplementary material for: Can paternal leakage maintain sexually antagonistic polymorphism in the cytoplasm?
Source: J Evol Biol. 2015 Feb 27;28(2):468–80. doi: 10.1111/jeb.12582 (PMC4413368; doi:10.1111/jeb.12582)
Supplement: Supplementary file 1 [file jeb0028-0468-sd1.pdf]

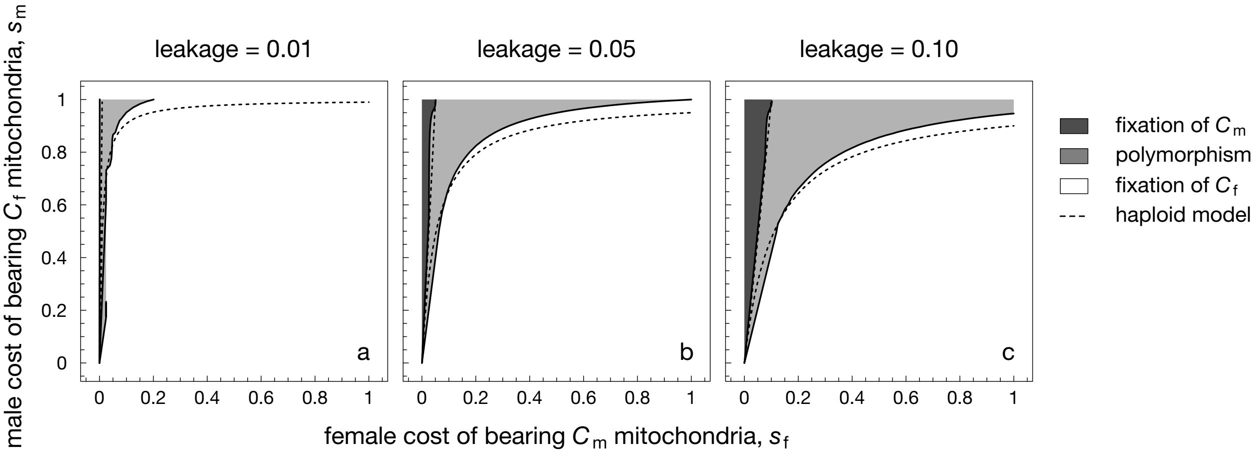

Figure S1:

Figure S1 Cytoplasmic polymorphism with multiple mitochondria per individual and the shape of male and female fitness functions is given by a linear function (additive). The model predicts polymorphism to be slightly less likely in comparison to the haploid model (dashed lines). This is because this model uses a conservative cut-off point of 0.05 to define polymorphism. Parameters: $M = 200$, $\mu = 1 \times 10^{-5}$.

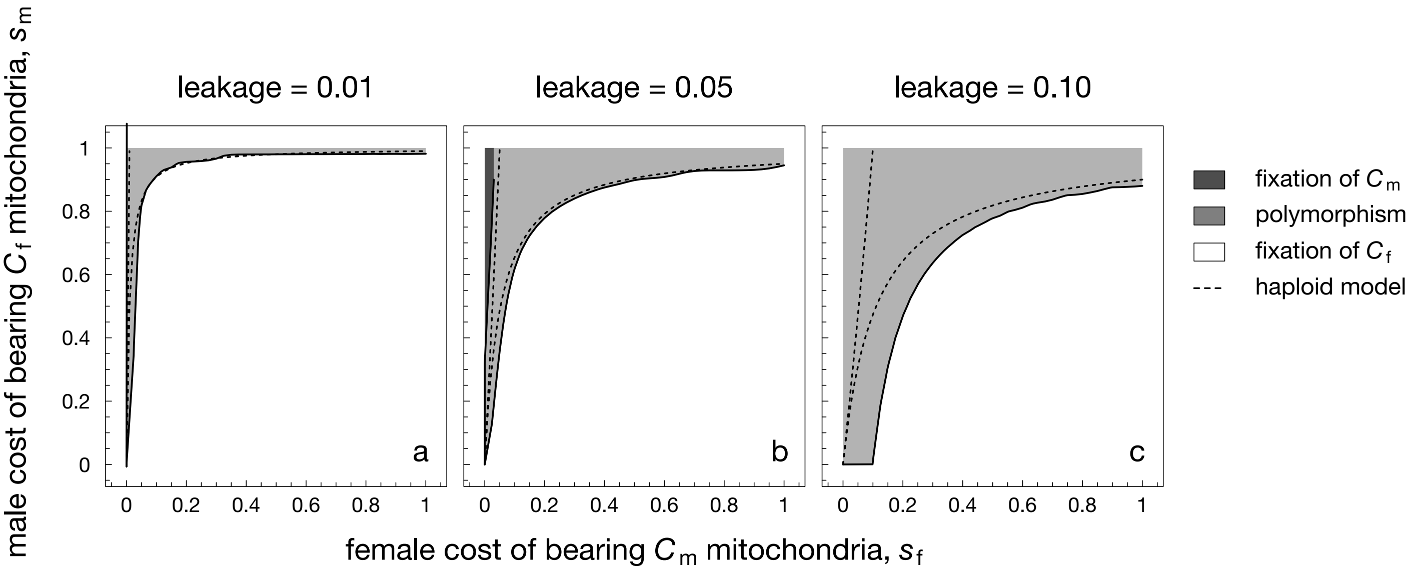

Figure S2:

Figure S2 Cytoplasmic polymorphism when multiple mitochondria per individual are present and the shape of male and female fitness functions is given by a sigmoidal function (see Figure 2D). The region in which a cytoplasmic polymorphism occurs is similar relative to the haploid model (dashed lines). The function of the sigmoidal is given by $w_{\mathrm{f}} = 1 - s_{\mathrm{f}} + s_{\mathrm{f}}\exp\left[-k(M-m)/M\right] / \left\{1+\exp\left[-k(n = M-m)/M\right]\right\}$ and $w_{\mathrm{m}} = 1 - s_{\mathrm{m}} + s_{\mathrm{m}}\exp\left[k(M-m)/M\right] / \left\{1+\exp\left[k(M-m)/M\right]\right\}$, where $k = 0.1$. Parameters: $M = 200$, $\mu = 1 \times 10^{-5}$, $B = 200$.

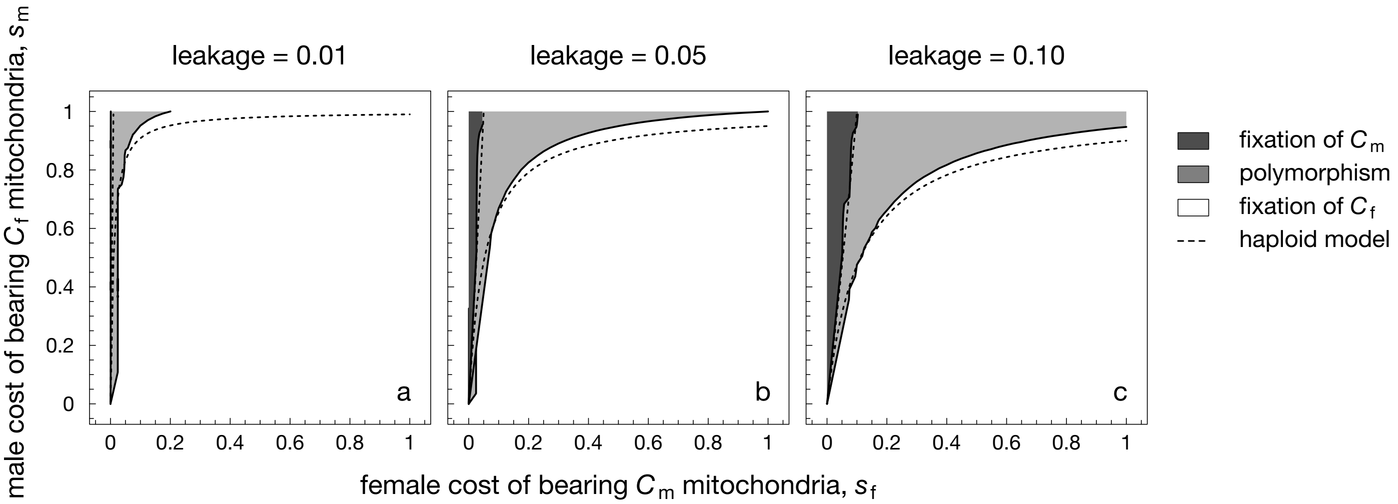

Figure S3:

Figure S3 Cytoplasmic polymorphism when the shape of male and female fitness functions is additive and the size of the bottleneck $B = 10$. Although the region of polymorphism is slightly smaller relative to the haploid model, this is due to the conservative demarcation of the region of polymorphism at $p = 0.05$ for the model in which each individual contains multiple cytoplasmic elements. A comparison with relative to Figure S1 (no bottleneck) shows that bottlenecks have little effect when fitness is additive. Parameters: $M = 200,\ \mu = 1 \times 10^{-5}, B = 10$.

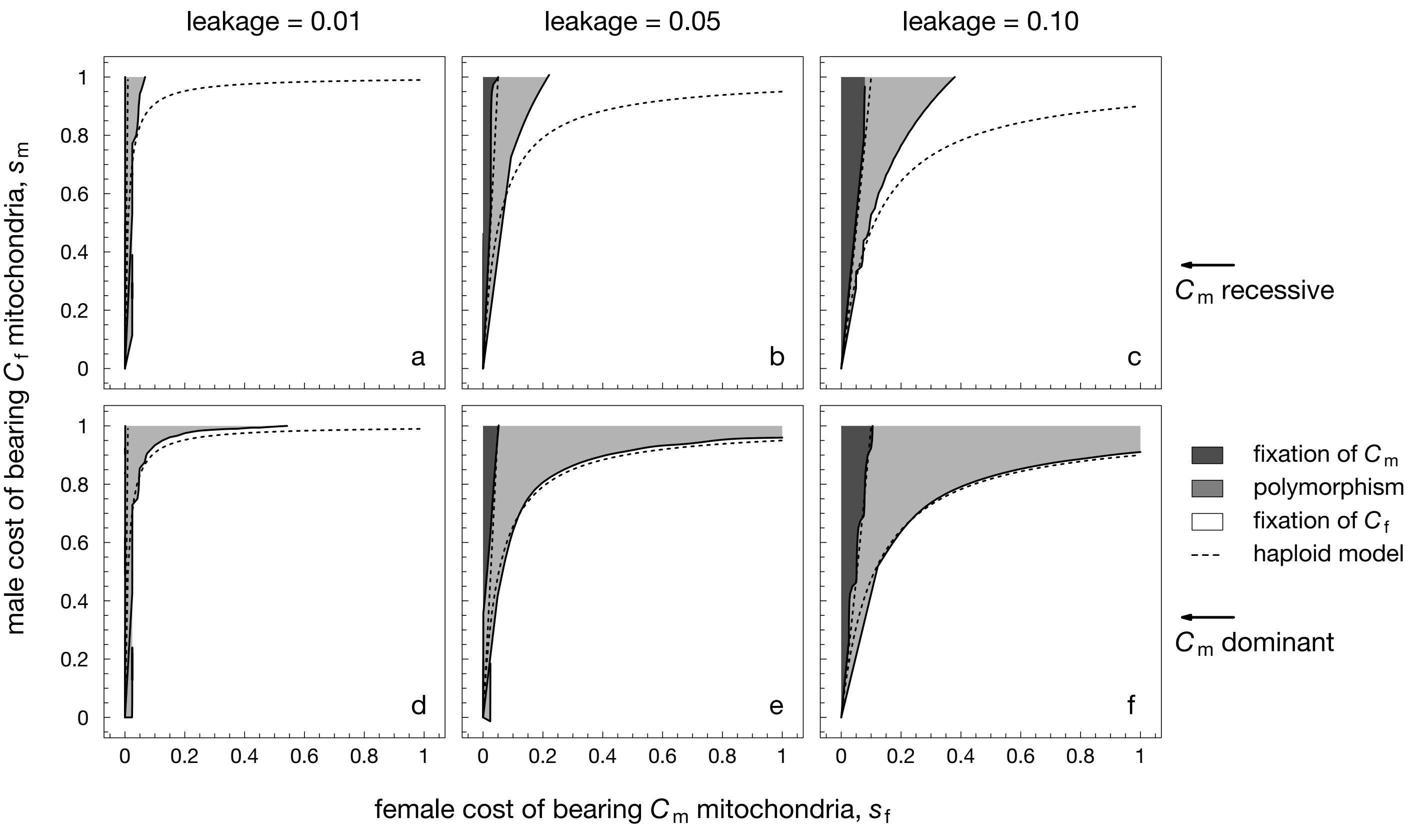

Figure S4:

635 **Figure S4** Cytoplasmic polymorphism when the shape of male and female fitness functions is given by a scenario of constant dominance (solid lines in Figure 2B,C) and the size of the bottleneck $B = 10$. Outcomes are similar to a scenario without bottlenecks in Figure 4. Parameters: $M = 200$, $\mu = 1 \times 10^{-5}, B = 10$.
