# Supplementary material for: Can paternal leakage maintain sexually antagonistic polymorphism in the cytoplasm?
Source: J Evol Biol. 2015 Feb 27;28(2):468–80. doi: 10.1111/jeb.12582 (PMC4413368; doi:10.1111/jeb.12582)
Supplement: Supplementary file 2 — Data S1 Model with multiple mitochondria. [file jeb0028-0468-sd2.pdf]

Supplementary Information to:

Can paternal leakage maintain sexually antagonistic polymorphism in the cytoplasm?

Bram Kuijper, Nick Lane & Andrew Pomiankowski

Model with multiple mitochondria

Following the model by Hadjivasiliou *et al.* (2012), we study a species with separate sexes, whose gametes contain multiple mitochondria. Let M denote the number of mitochondria present in each cell and let j describe the number of mitochondria which carry a C_m-allele ($j \in \{0, 1, \ldots, M\}$), implying that $M - j$ mitochondria in the same cell carry the C_f-allele. The frequency of cells which contain j C_m-mitochondria prior to reproduction is denoted by $p_{0,\mathrm{f}}(j)$ in females and $p_{0,\mathrm{m}}(j)$ in males.

S1.1 Mitochondrial mutation

Analogous to recent models of sexually antagonistic polymorphisms in nuclear genomes (e.g., Connallon & Clark, 2012; Mullon *et al.*, 2012), we assume the presence of recurrent mitochondrial mutation. Specifically, each mitochondrial allele mutates with probability μ. Hence, in order to compute the relative frequency of each genotype $p_{x,1}(j)$ in sex $x \in \{\mathrm{m}, \mathrm{f}\}$ subsequent to mutation, we need to assess the probability of mutation from all possible other genotypes $k \neq j$. Writing the genotype frequencies $p_x(j)$ in vector form, $\mathbf{p}_0 = [p_\mathrm{m}(0), p_\mathrm{m}(1), \ldots, p_\mathrm{m}(n), p_\mathrm{f}(0), p_\mathrm{f}(1), \ldots, p_\mathrm{f}(M)]^\mathrm{T}$ (where T denotes transposition) , we have

$$\mathbf{p}_1 = \mathbf{U}\mathbf{p}_0, \tag{S1}$$

where $\mathbf{U} = \begin{bmatrix} \mathbf{u} & \mathbf{0} \\ \mathbf{0} & \mathbf{u} \end{bmatrix}$ is a $2(M+1) \times 2(M+1)$ block diagonal matrix of which entry $u(k, \ell)$ describes the probability of mutating from genotype ℓ to genotype k. Following Hadjivasiliou *et al.* (2012), we assume that each mitochondrial gene only mutates once during an individual's lifespan. Hence, given a probability

of mutation of μ for each gene, the entry $u_{k\ell}$ is given by

$$u_{k\ell} = \sum_{g_2=0}^{M-\ell} \sum_{g_1=0}^{\ell} \text{Binom}(x = g_1, \mu, \ell)\, \text{Binom}(x = g_2, \mu, M-\ell)\, \delta_{\ell - g_1 + g_2 = k}, \tag{S2}$$

where Kronecker's delta indicates that only those combinations are incorporated which result in the desired number of k C_m alleles.

S1.2 Fitness

See main text for a description of the fitness effects.

Recursions The frequencies $p_{2,\text{m}}(j)$ and $p_{2,\text{f}}(j)$ of mitochondrial type j after selection in males and females respectively subsequent to selection are given by

$$p_{2,\text{m}}(j) = \frac{w_\text{m}(j) p_{1,\text{m}}(j)}{\bar{w}_\text{m}} \tag{S3a}$$

$$p_{2,\text{f}}(j) = \frac{w_\text{f}(j) p_{1,\text{f}}(j)}{\bar{w}_\text{f}}, \tag{S3b}$$

where $\bar{w}_x = \sum_{j=0}^{n} p_{1,x}(j) w_x(j)$ is the mean fitness for sex x.

S1.3 Bottlenecks

First, M mitochondria are sampled without replacement down to B mitochondria. Subsequently, in the newly divided cell, mitochondria are randomly selected with replacement to divide again until a number of M is restored. Let the vector $\mathbf{p}_3$ denote the frequency distribution of mitochondrial genotypes after the bottleneck. We then have

$$\mathbf{p}_2 = \mathbf{A}\mathbf{p}_2$$

where $\mathbf{A} = \begin{bmatrix} \mathbf{b} & \mathbf{0} \\ \mathbf{0} & \mathbf{b} \end{bmatrix}$ is again a block diagonal matrix with entry $b_{k\ell}$ given by

$$b_{\ell \to k} = \sum_{h_2=0}^{M} \sum_{h_1=0}^{B} P_{B_1}(\ell \to h_1) P_{B_2}(h_1 \to k)$$

where $P_{B_1}(\ell \to h_1)$ is the transition probability from a cell that contains ℓ alleles preceding the bottleneck and h_1 alleles afterwards, according to a hypergeometric distribution

$$P_{\text{Bottleneck}}(\ell \to h_1) = \frac{\binom{\ell}{h_1}\binom{M-\ell}{B-h_1}}{\binom{M}{B}}$$

wheres $P_{B_2}(h_1 \to k)$ reflects the transition probability from h_1 C_{m} alleles following the bottleneck to k alleles after re-sampling back to M mitochondria. This is given by a binomial distribution with

$$P_{B_2}(h_1 \to k) = 1, \quad \text{when } k = 0 \text{ and } h_1 = 0$$
$$P_{B_2}(h_1 \to k) = \binom{M}{k}\left(\frac{h_1}{B}\right)^k \left(\frac{B-h_1}{B}\right)^{M-k}$$

S1.4 Meiosis I and II

Meiosis I In meiosis I, each mitochondrion is duplicated and then two daughter cells are formed by random segregation. Each receives a random sample of the $2M$ mitochondria present in the parent cell. Let $a_{k\ell}$ reflect the probability that a mother cell with ℓ mitochondria gives rise to a daughter cell with k mitochondria. Again we have a similar block matrix dynamic as previously

$$\mathbf{p}_5 = \mathbf{E}_1 \mathbf{p}_4,$$

in which element $\varepsilon_{1,k\ell}$ in the diagonal blocks of the matrix $\mathbf{E}_1$ is given by

$$\varepsilon_{1,k\ell} = \frac{\binom{2\ell}{k}\binom{2(M-\ell)}{M-k}}{\binom{2M}{M}}.$$

Meiosis II In meiosis II, each newly formed diploid daughter cell now divides into two gametes, each containing $M/2$ mitochondria through random segregation. The vector $\mathbf{p}_6$ is the distribution of mitotype numbers after meiosis II, which contains zeros for frequencies $p_f(j)$ and $p_m(j)$ where $j > M/2$. Given the dynamic $\mathbf{p}_6 = \mathbf{E}_2\mathbf{p}_5$, the transition matrix $\mathbf{E}_2$ is given by

$$\mathbf{E}_2 = \begin{bmatrix} \mathbf{e} & \mathbf{0} \\ \mathbf{0} & \mathbf{0} \\ \mathbf{0} & \mathbf{e} \\ \mathbf{0} & \mathbf{0} \end{bmatrix}$$

with elements

$$\varepsilon_{2,k\ell} = \frac{\binom{\ell}{k}\binom{M-\ell}{M/2-k}}{\binom{M}{M/2}}$$

S1.5 Syngamy

Uniparental inheritance We assume that the mitochondrial genome of one parent is discarded, and that of the other is doubled through sampling with replacement. The distribution of genotypes is then given by the vector $\mathbf{p}_7 = \mathbf{S}\mathbf{p}_6$, where

$\mathbf{S} = \left[\begin{smallmatrix} \mathbf{0} & \mathbf{s} \\ \mathbf{0} & \mathbf{s} \end{smallmatrix}\right]$ .Elements of the sub-matrices $\mathbf{s}$ are given by

$$s_{k\ell} = \binom{M}{k}\binom{2\ell}{M}^k\binom{M-2\ell}{M}^{M-k}.$$

Paternal leakage Given paternal leakage, $0 < \pi < \frac{M}{2}$ denotes the number of mitochondria inherited from the father, so $0 < 1-\pi < \frac{M}{2}$ mitochondria are inherited from the mother. After syngamy, the resulting set of $M/2$ mitochondria is duplicated until the required number of M zygotic mitochondria is reached. Consider a sperm cell that has a number of r_m C_m mitochondria, whereas the egg contains r_f C_f mitochondria. Let $0 < j_m < \pi$ be the number of C_m mitochondria among those mitochondria that are inherited through sperm. Similarly, let $0 < j_f < 1-\pi$ be the number of C_m mitochondria that are inherited

through the egg, where the total contribution of C_m mitochondria is $k = j_\mathrm{f} + j_\mathrm{m}$. Hence, we have

$$p_7(k) = \sum_{j_\mathrm{m}=0}^{\pi} \sum_{r_\mathrm{m}=0}^{M/2} \sum_{r_\mathrm{f}=0}^{M/2} p_{\mathrm{m}6}(r_\mathrm{m})\, p_{\mathrm{f}6}(r_\mathrm{f})\, \epsilon(r_\mathrm{m} \to j_\mathrm{m}, \pi)\, \epsilon(r_\mathrm{f} \to k - j_\mathrm{m}, 1-\pi)$$

where $\epsilon(r \to j, x)$ reflects the probability that a gamete cell containing a number of r C_m-mitochondria contributes $0 \leq j \leq x$ C_m mitochondria to the zygote. Hence, $\epsilon(r \to j, x)$ is given by

$$\epsilon(r \to j, x) = \frac{\binom{r}{j}\binom{M/2-r}{x-j}}{\binom{M/2}{x}}.$$

Subsequently, the $M/2$ mitochondria are duplicated by re-sampling until the required number of n zygotic mitochondria is achieved, or

$$p_8(k) = \sum_{z=0}^{M/2} p_7(z) \binom{M}{k} \left(\frac{2z}{M}\right)^k \left(\frac{M-2z}{M}\right)^{M-k}$$

S1.6 Analysis

We numerically iterated recursions for $\mathbf{p}$ by coding a system of $2(M+1) \times 2(M+1)$ recursion equations in C. We assumed that equilibria are reached when the differences in mitotype frequencies between subsequent time steps are smaller than 1×10^{-7}.
